# In Silico-Based Experiments on Mechanistic Interactions between Several Intestinal Permeation Enhancers with a Lipid Bilayer Model

**DOI:** 10.1021/acs.molpharmaceut.1c00689

**Published:** 2021-12-16

**Authors:** Rosita Kneiszl, Shakhawath Hossain, Per Larsson

**Affiliations:** † Department of Pharmacy, Uppsala University, Husargatan 3, Uppsala 751 23, Sweden; ‡ The Swedish Drug Delivery Center (SweDeliver), Uppsala University, Husargatan 3, Uppsala 751 23, Sweden

**Keywords:** oral peptide drug delivery, intestinal permeation enhancers, molecular dynamics (MD) simulations, medium chain fatty acids, salcaprozate sodium (SNAC), sucrose monolaurate

## Abstract

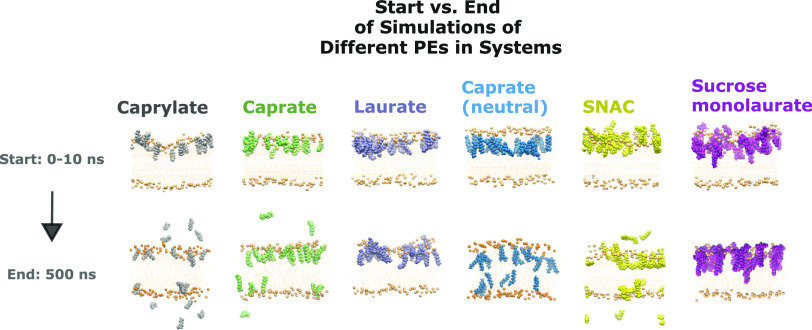

Oral administration of drugs is generally considered convenient and patient-friendly. However, oral administration of biological drugs exhibits low oral bioavailability (BA) due to enzymatic degradation and low intestinal absorption. A possible approach to circumvent the low BA of oral peptide drugs is to coformulate the drugs with permeation enhancers (PEs). PEs have been studied since the 1960s and are molecules that enhance the absorption of hydrophilic molecules with low permeability over the gastrointestinal epithelium. In this study, we investigated the impact of six PEs on the structural properties of a model membrane using molecular dynamics (MD) simulations. The PEs included were the sodium salts of the medium chain fatty acids laurate, caprate, and caprylate and the caprylate derivative SNAC—all with a negative charge—and neutral caprate and neutral sucrose monolaurate. Our results indicated that the PEs, once incorporated into the membrane, could induce membrane leakiness in a concentration-dependent manner. Our simulations suggest that a PE concentration of at least 70–100 mM is needed to strongly affect transcellular permeability. The increased aggregation propensity seen for neutral PEs might provide a molecular-level mechanism for the membrane disruptions seen at higher concentrations in vivo. The ability for neutral PEs to flip-flop across the lipid bilayer is also suggestive of possible intracellular modes of action other than increasing membrane fluidity. Taken together, our results indicate that MD simulations are useful for gaining insights relevant to the design of oral dosage forms based around permeability enhancer molecules.

## Introduction

Scientists have tried to enable oral administration of protein and peptide-based therapeutics ever since insulin^1^ was discovered 100 years ago for the treatment of diabetes mellitus. Oral administration of drugs is generally considered as more convenient and more patient-friendly than other routes of administration. Successful oral administration of biological drugs, such as peptides, as well as small hydrophilic/hydrophobic drug molecules with low permeability is difficult, with various factors contributing to low absorption.^2^ These are mainly due to inherent physicochemical properties of peptides and to the physiology of the gastrointestinal (GI) tract. Peptide molecules are degraded by the acidic environment in the stomach and by the enzymes present in the stomach and in the lumen of the GI tract.^2,3^ For this reason, the majority of therapeutic peptide drugs are administered parenterally.^1,4^ Although technology has become much better over the last decades, the risk of discomfort and pain^5^ from the injection remains, as also the risk of a decrease in patient compliance.^6,7^ In recent times, technologies have been developed to improve the peptide-based formulation and peptide structure against enzymatic degradation. Despite this, peroral peptides are in general too large and too hydrophilic to permeate the intestinal epithelium.^8^ Therefore, coformulation of peptides as well as poorly permeable drug molecules with intestinal permeation enhancers (PEs) could be an approach to enable their intestinal epithelial permeability.^9−11^ PEs (also termed absorption enhancers) have been studied since the 1960s^12^ and are molecules that enhance the absorption of hydrophilic molecules with low permeability over the GI epithelium, including peptides,^11^ and for instance, vitamin B12.^13^ A thorough description of PEs can be found in the article by Maher et al.^10^ Despite the extensive information available regarding PEs, there is incomplete knowledge regarding the precise molecular mechanisms of action of PEs.^14^ Furthermore, how different PEs affect the properties of lipid bilayers is not completely understood.

PEs can be categorized as tight junction selective and/or membrane perturbing.^15^ One class of molecules often studied as PEs are the sodium salts of medium chain fatty acids (MCFAs) such as laurate (C_12_), caprate (C_10_), caprylate (C_8_), and the caprylate derivative sodium N-(8-[2-hydroxybenzo]amino) caprylate (Salcaprozate sodium/SNAC) in the Eligen carrier technology^16^ by Emisphere Technologies, New Jersey, USA.

Caprate is present in dairy products and in various oils^12^ and has a food additive status. Caprylate is also safely used in food.^17^ SNAC has a GRAS (Generally Recognized as Safe) status and is a component of the U.S. Food and Drug Agency (FDA)-approved Eligen Vitamin B12.^13,18^ Caprate is deprotonated or sodium salt of capric acid, and it is a mild surfactant. The modes of action are believed to be both by transcellular perturbation such as altering the fluidity of the plasma membrane and mucosal perturbation at a higher concentration and by direct and indirect paracellular mechanisms at a lower concentration.^10,19,18^ Caprylate is also deprotonated or sodium salt of caprylic acid, and as caprate, it is a mild surfactant and assumed to fluidize the plasma membrane.^14^ Marketed products using these PEs include the FDA-approved (June 2020) Mycapssa, developed by Chiasma (Needham, Massachusetts, USA), for the treatment of acromegaly. This is an oral capsule of the somatostatin analogue octreotide in combination with the transient permeability enhancer (TPE) technology—an oily suspension of different pharmaceutical excipients, including caprylate. In September 2019, FDA, followed by the European Medicines Agency in April 2020, approved Rybelsus.^20,21^ It is developed by Novo Nordisk A/S (Bagsvaerd, Denmark) and studied in the PIONEER clinical trials for the treatment of adults with the metabolic disease type 2 diabetes mellitus. Rybelsus is a tablet consisting of the glucagon-like peptide-1 receptor agonist semaglutide in coformulation with SNAC.^22^ Insulin coformulation with SNAC^23^ and 4-CNAB,^24^ respectively, has also been studied, and a lowering in the glucose effect in patients was observed in both studies. The modes of action of SNAC are described in the research paper of Buckley et al.^22^ and in the reviews of Twarog et al.^18^ and of Bucheit et al.,^25^ where the mechanisms are explained to be by the prevention of destruction (such as elevation in gastric pH and inhibition of gastric digestive enzyme pepsin) and the increase in lipophilicity of semaglutide, which enable passive transcellular permeation of the gastric membrane. According to Buckley et al.,^22^ the permeation-enhancing effect of the ortho isomer of SNAC, *o*-SNAC, was distinctly less in comparison to that of SNAC. The difference in effect between *o-*SNAC and SNAC highlights the importance of understanding the molecular-level effects exerted by these molecules.

The nonionic surfactant molecule sucrose monolaurate is also indicated by McCartney et al.^26^ as a possible PE. Its mechanism of action is described to be indirect, by the opening of tight junctions via membrane perturbation.

The MCFA laurate (C_12_) is believed to have a paracellular effect at lower concentrations and a mucosal damaging effect at higher concentrations. However, as far as we know, laurate as a PE has not been tested clinically, as opposed to the other MCFAs.

The phosphatidylcholine 1-palmitoyl-2-oleoyl-sn-glycero-3-phosphocholine (POPC) molecule is one of the most abundant phospholipids in eukaryotic cell membranes, and it is often used in model systems to represent a bilayer. Oral drug absorption over lipid bilayers can be studied in in vitro models using transwell experiments with cell lines, as for example the human colon carcinoma cell line Caco-2,^12,27^ or with the Everted sac model.^28,29^ Absorption can also be studied in ex vivo models with different segments of mouse, rat, rabbit, or human intestinal region tissue mounted in, for example, an Ussing chamber model,^30,12^ in a Franz cell model,^12^ or in an organ culture model of intestinal mucosal explants,^15^ as well as with cell-imaging tools combined with biophysical methods.^18^ The human colorectal adenocarcinoma cell line, Caco-2,^31,32^ can be used as an in vitro transport model system to study permeability of the small intestinal epithelia.^31^ During cultivation, the Caco-2 cell line differentiates into cell monolayers and has a morphology resembling the small intestinal epithelium^32^ and permeation patterns resembling the colonic epithelia.^31^ However, a disadvantage with these models is that the level of detail is limited. A comparison in the general mechanisms of action between SNAC and sodium caprate^28^ is reported in Twarog et al., in which the authors suggest common mechanisms of action with indirect changes in the tight junctions resulting from membrane perturbation, as well as direct membrane effects. However, the molecular-level details remain elusive.

**Figure 1:**
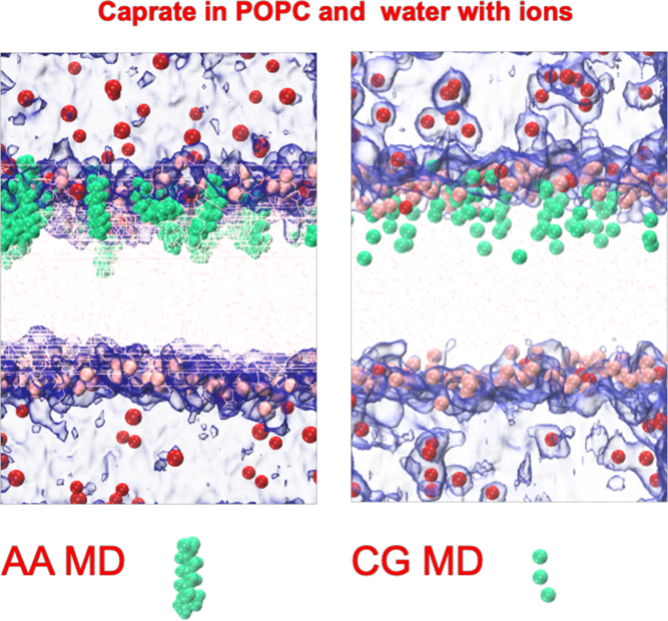
Illustration of the difference in the level of details of a molecule of sodium caprate (in green) between all-atom (AA) molecular dynamics (MD) simulations (to the left) and coarse-grained (CG) MD simulations (to the right). The sodium chloride ions are in red, the phosphate headgroups of the POPC molecules are in pale pink, the dots in the center are the lipid tails of the POPC molecules, and the water is in faded blue. A single caprate molecule represented in both AA and CG is also added in the lower panel.

Molecular dynamics (MD) is a computational simulation technique (in silico experiments) that allows for the study of interactions on an atomic (all-atom, AA) level. Newton’s equations of motion are used to describe the behavior of a simulated system in terms of the motion of individual atoms as a function of time. MD simulations can advantageously be used in combination with wet laboratory experiments in order to complement and complete such experiments. Of relevance here, they have been used to study, for instance, molecular interactions of various PEs,^33^ nanoparticles,^34^ membrane fluidity, and membrane structural properties.^35−37^ For more information on membrane studies with MD simulations, see the review article of Moradi et al.^38^ One of the limitations with AA MD simulations is that they are computationally demanding and typically can only be applied to the study of the process on very short time and length-scales. To alleviate some of these shortcomings, simulations can be accelerated using coarse-grained (CG)^39^ MD by assembling several atoms into larger, CG beads. The obvious advantage with CG MD is that it saves time, but a potential limitation is the loss of detail (Figure 1).

To date, the typical development process of pharmaceutical dosage forms has not fully embraced MD simulations, meaning that there is a lack of detailed understanding of how formulation components (such as PEs) behave and interact with the intestinal milieu. The aim of this study was to take one step toward more knowledge-based decisions about which components (PEs) to include in a dosage form for oral peptide administration by investigating the effects of intestinal PEs (believed to have some degree of a transcellular mode of action) on the structural and dynamical properties of the lipid POPC bilayer.

## Methods

All simulations were performed with Gromacs 2018^40^ using the Charmm36 force field.^41,42^ Systems were built with the web-based tool CHARMM-GUI^43,44^ Membrane Bilayer Builder.^45−47^ Each system contained a POPC membrane with a certain type of PE at different concentrations. One system with only POPC was also created. All systems contained 64 POPC molecules in each leaflet of the membrane. In the systems with PEs, six different PEs were used (Table 1), where, again, all included PEs are believed to have a multimodal mechanism of action.^22,26,14^ Each type of PE was inserted into one of the leaflets in each system at different concentrations before the start of the simulations, when creating the systems with the web-based tool CHARMM-GUI’s Membrane Bilayer Builder.^43,45^ These number concentrations were varied in the range of approximately 5–35% of the total number of lipid molecules present in the leaflet based on number (e.g., 5% of 64 POPC molecules in the upper leaflet equals in 3 PE molecules inserted), corresponding to about 20–160 mM (Supporting Information Table S1). The average length during the simulations of the *x*- and *y*-box vectors of the simulated systems was 6.60 nm, and that of the *z*-box vector was 9.35 nm on average.

**Table 1:**
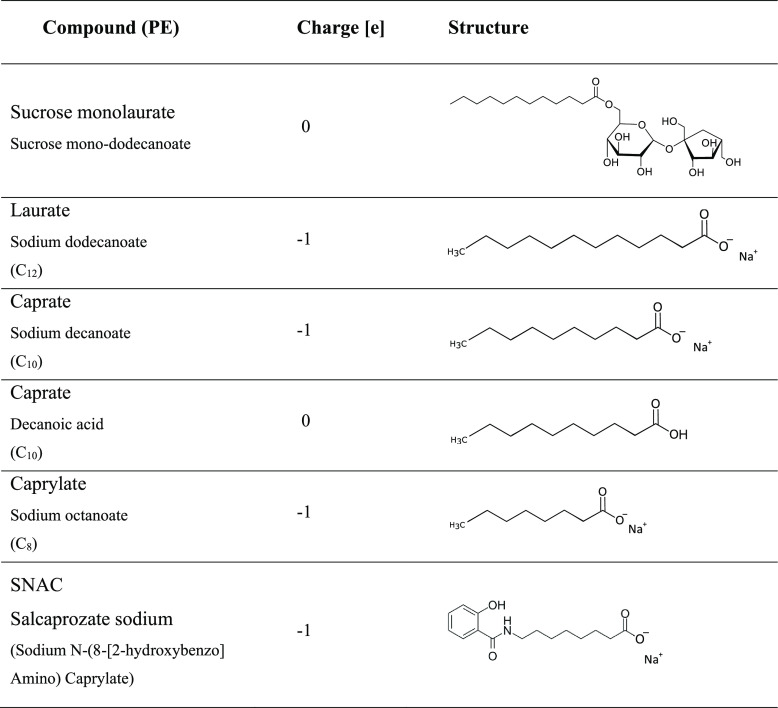
Names, Charged State, and Chemical Structures of the Six Different Permeation Enhancers (PEs) Used in This Study

A concentration of 150 mM sodium chloride, based on the volume of water of the system, was added to each system to represent physiological salt concentration. Simulations were performed at 37 °C (310.15 K) to mimic body temperature and at an average of 1 bar with semi-isotropic pressure coupling using the Parrinello–Rahman barostat^48^ (reference pressure = 1 bar), coupling time constant = 5 ps, and compressibility = 4.5e^–5^ bar^–1^. Electrostatic interactions were treated with particle-mesh Ewald with a short-range cut-off of 1.2 nm, and van der Waals interactions were switched off using a force-based cut-off between 1.0 and 1.2 nm. Bonds involving hydrogens were constrained using the LINCS algorithm. After energy minimization and system equilibration, the final production run was performed for 500 ns for each system using a 2-fs timestep.

### CG MD Simulations

To run CG MD simulations of caprate, caprylate, and SNAC with negatively charged headgroups, a membrane-only system with 64 POPC molecules in each leaflet was first created with the Martini Maker^49,50^ Bilayer Builder in the CHARMM-GUI^43^ tool. For each case, 22 PE molecules were inserted in one leaflet to represent 35% of the total number of lipid molecules present in the leaflet. The simulations were performed using the Martini force field.^51,52^

### Generation of PE Topologies

PE molecular topologies for laurate, caprate, and caprylate are readily obtained from the Charmm36 force field files. First, the topology for laurate was generated in the CHARMM-GUI online tool,^43^ and topologies for caprate and caprylate were created by removing the appropriate number of atoms/bonds/angles. The input topology for SNAC was created starting with an automated parameterization process using the Charmm General Force Field (CGenFF) 1.0.0 program,^53,54^ resulting initially in a topology with low penalties (<10), but with penalty scores between 10 and 50 for two dihedrals. A subsequent refinement of these dihedral potentials was therefore carried out with the fftk toolkit in visual molecular dynamics (VMD).^55^ The topology for sucrose monolaurate was generated by combining the C12 topology with that of the appropriate sugar moiety from the Charmm force field files. The CG caprylate and caprate topologies were developed and validated as described in Hossain et al.^33^ The CG SNAC topology used in this study was developed and validated in the work of Hossain et al.^37^

### Analyses

All simulated systems were analyzed over the entire trajectory with respect to how different types and concentrations of PE molecules might perturb lipid bilayers and affect structural properties, as well as the dynamics of the interactions. To start, because all PEs were initially inserted into the membranes, we estimated the number of PE molecules that leave over time (expulsion events) from the leaflet, taken to represent the apical side of intestinal membranes during the simulations. To count the number of expulsion events of PE molecules, the gmx select tool of Gromacs 2016 was used, with an expulsion defined as a PE molecule not being within a distance of 0.9 nm of any of the phosphate atoms of the POPC molecules in the leaflet. Note that the expulsion events were only considered if the PE molecules were expelled from the membrane into the water. Once the PE molecules moved from one leaflet to another, it was considered as a flip-flop event. PE flip-flop events were investigated using a similar approach, and also using the methodology and code presented in the work of Zawada et al.^56^ Due to the use of periodic boundary conditions, PE molecules can become part of the other leaflet without traversing the lipid bilayer. To estimate true flip-flop events, we took care to ensure that the molecules actually flipped and did not cross the periodic boundary.

Changes in different structural properties of the POPC membrane such as area per lipid headgroup (APL), membrane thickness, overall order parameter (deuterium order parameter, DOP), and lateral diffusion coefficients of POPC molecules were also calculated. The APL was calculated by multiplying the length of the x-axis of the box with the length of the y-axis. This number was divided by the number of the total amount of lipid molecules in the same leaflet (*n* = 64) that the PEs were inserted into. No flip-flop event of the POPC molecules was present in any of the simulations. To calculate the membrane thickness, we used an open-source software called Fatslim.^57^ The thickness measurement was defined by selecting the phosphate atoms of the POPC molecules in each leaflet. The detailed calculation procedure can be found in the article by Buchoux.^57^

The overall order parameter (DOP) provides information regarding the alignment of the lipid chains of the POPC molecule along the bilayer normal (*z*) direction. A value of 1 (*S*_CD_ = 1) constitutes perfect alignment, while the opposite is valid for a value of −0.5 (anti-alignment). A value of 0 corresponds to a random orientation of the lipid chains in relation to the bilayer normal. The overall order parameter was calculated using the gmx order tool. Lateral diffusion, *D*_L[POPC]_, is a measurement of the movement of the POPC molecules in the *x*- and *y*-planes, with or without the additional PEs present. With the gmx msd-tool, the lateral diffusion was determined for the leaflet with the PEs present, using the phosphate atom in the POPC molecule as the reference atom.

The permeation of water molecules into the bilayer was quantified by counting water molecules with the gmx select tool. In order to capture any penetration of water molecules deep into the hydrophobic membrane region, the three last carbons in the POPC lipid tails and their hydrogens were selected to represent the central membrane region. All water molecules within 0.5 nm of this part of the acyl chains were then counted, as a measure of how permeable the membrane was to water molecules in the presence of the different types of PEs.

The normalized fractional interactions were calculated as the relative number of contacts between the POPC and PE molecules present in the membrane leaflet, with a correction for the total number of molecules of each kind present in the leaflet.^58,59^ Such a measure of fractional interactions was previously used to characterize the degree of lipid associations in complex membranes,^59^ phase separation,^60^ and lipid–peptide interaction.^59^ For a two-component system such as the POPC-PE system in our study, a fraction of 0.5 indicates a randomly mixed membrane. The total number of contacts was obtained using the gmx mindist tool in Gromacs with a cut-off distance of 0.9 nm for each component. The VMD program was used to generate the simulation snapshots images.^61^

### Umbrella Sampling Simulations

Umbrella sampling (US) simulations were performed to compute the potential of mean force (PMF) profiles for pulling caprate, caprylate, and SNAC molecules from the membrane center to the water phase using both the AA CHARMM36 and CG Martini force fields. To perform the US simulations, a series of configurations were generated along the reaction coordinate, which in this case was the distance from the membrane center to the bulk water phase. Twenty configurations separated at a distance of 0.1 nm along the reaction coordinate were generated for each case. Each configuration that served as the starting point for the US simulations was energy-minimized and equilibrated followed by a production run for 20 ns. The weighted histogram analysis method (WHAM) implemented in Gromacs (gmx wham) was used to extract the PMF along the reaction coordinate from the US simulations.^62^

## Results and Discussion

### Interactions of PEs with the Model Cell Membrane

**Figure 2:**
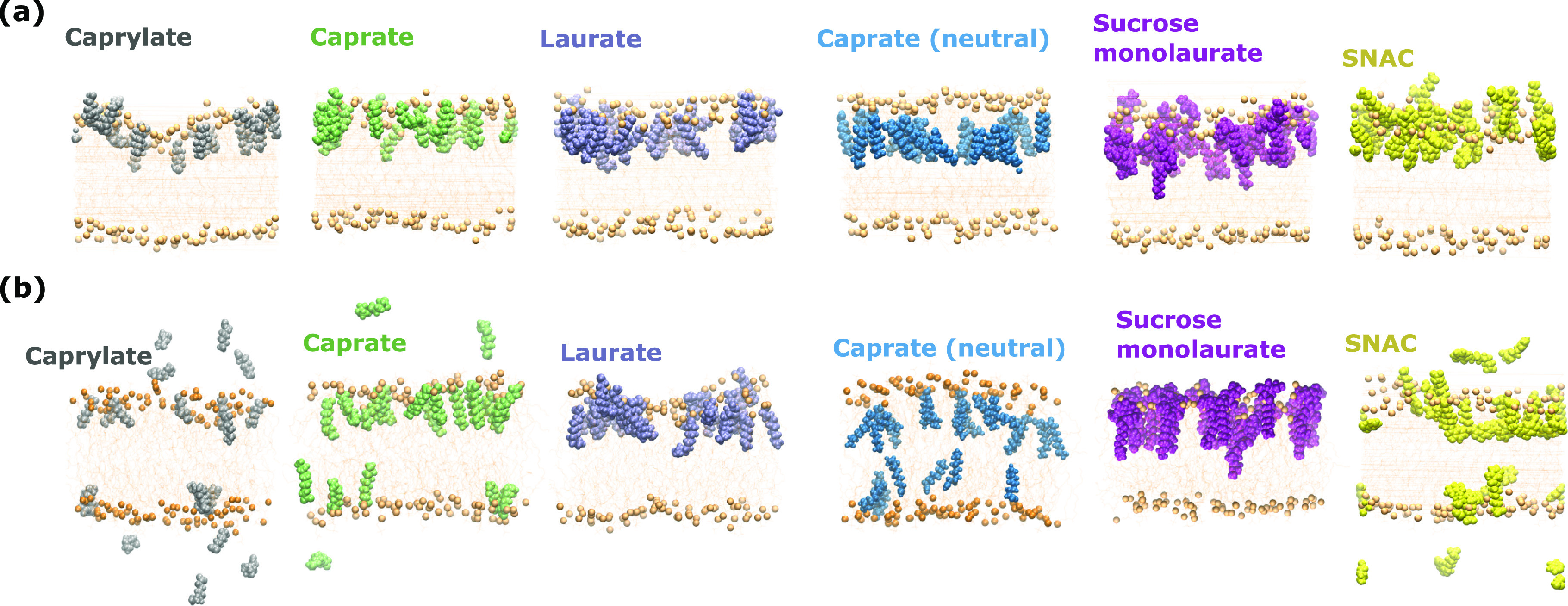
Interactions of different PEs with the POPC membrane. Snapshot of (a) initial, representative states, up to 10 ns and (b) final (after 500 ns simulations) system configurations of the POPC membrane with 35% concentrations of the PEs. The pale orange spheres are the phosphate headgroups of the POPC molecules, and the even paler orange in between the upper and lower rows with spheres represents the phospholipid acyl chains of the POPC molecules.

To investigate interactions between PE molecules and model cell membranes, we have performed both AA and CG simulations by initially placing six different types of PEs inside POPC membranes. The PEs were placed only in one of the leaflets, taken to represent the apical side of the small intestinal epithelium. The number concentration of the PEs was varied in the range of approximately 5–35% (24 to 164 mM) of the total number of lipid molecules present in the leaflet. Initial snapshots of the six different PEs used in this study, at 35% concentration, are shown in Figure 2A.

At the beginning of the simulation (at 0 ns), all the PEs remained near the headgroup region of the membrane POPC molecules. However, during the course of the simulations, different interaction patterns with the membrane were observed for different PEs. The PEs with relatively long tails/chain lengths, such as sucrose monolaurate and laurate, were found to remain mostly in the same membrane leaflet (i.e., no expulsion or flip-flop events occurred) for the complete 500 ns long simulations. However, for SNAC, caprylate, and caprate (both with negatively charged and neutral headgroups), expulsion or flip-flop of a number of molecules from the membrane leaflet occurred. Final snapshots from the simulations consisting of PEs at 35% concentrations are presented in Figure 2B for each PE. It is evident that a number of caprylate, caprate with negatively charged headgroups, and SNAC molecules have moved out to the water phase from the membrane leaflet, while a number of caprate molecules with neutral headgroups changed their location from one leaflet to another through flip-flop events. During the simulations, some molecules also crossed the periodic boundary of the system and became incorporated into the other (lower) leaflet. For a real intestinal epithelium, such events would not occur. However, in our simulations, we consider such an occurrence only as an expulsion event. It was not considered as a flip-flop event if a molecule crossed the periodic boundary and got incorporated into the other leaflet.

**Figure 3:**
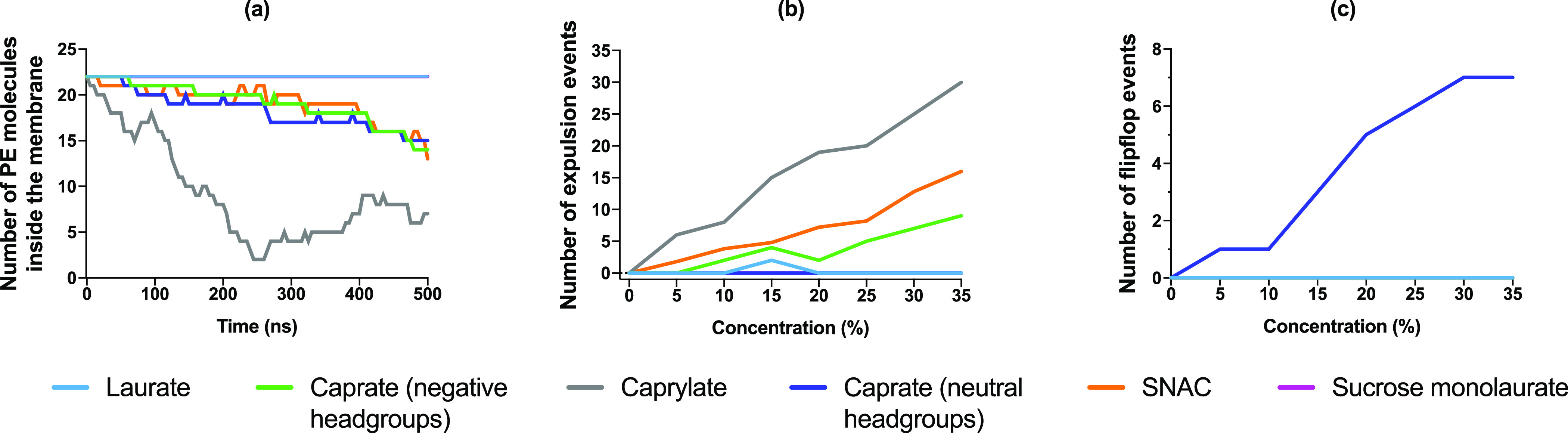
PE interaction with the model POPC membrane. (a) Number of PE molecules that remain incorporated in the initial leaflet (upper leaflet) of the membrane when 35% concentration of PEs was added in each system and (b) number of expulsion events occurred during the simulation for different PEs and (c) number of flip-flop events.

The variation in the number (concentration) of PE molecules that remained in the initial leaflet during the simulations for 35% PE concentration is presented in Figure 3A. The profile for caprylate shows a reduction with approx. 68% of PE molecules were expelled from the membrane leaflet. The profiles for caprate and SNAC showed about 36 and 40% reduction in PE molecules in the membrane leaflet, respectively. The variation of the PE molecules in the membrane for all other concentrations are presented in Figure S1. In that figure, the number of negatively charged sodium caprylate molecules which are expelled from the membrane seemed to reach a plateau after ∼200 ns, preceded by a steep, rapid, and concentration-dependent decrease in the number of molecules remaining in the membrane. A longer simulation of negative sodium caprate could tell if those molecules also would reach a plateau. Caprate molecules with neutral headgroups changed leaflet through flip-flopping, and in contrast to the other PEs, we did not observe the presence of such molecules in the water phase and there was also no crossing event through the periodic boundary. The occurrence of flip-flop events in the membrane for fatty acid molecules with a neutral headgroup was also observed in the past in both experimental and computational studies.^63,64^ The total number of expulsion events and flip-flop events for each PE at various concentrations are presented in Figure 3B,C, respectively. An increasing number of expulsion or flip-flop events with increasing concentration for caprylate, caprate, and SNAC molecules can be observed, along with occasional expulsion events occurring for laurate and no expulsion or flip-flop events for sucrose monolaurate molecules (Figure 3B). For the caprate molecules with neutral headgroups, an increasing number of flip-flop events with increasing PE concentration was also observed (Figure 3C).

In an earlier study by Hossain et al.,^37^ CG MD were used to reveal the inability of SNAC molecules to be incorporated deep into the membrane. These CG MD simulations also showed that caprate and caprylate, once incorporated inside the membrane, were not expelled from the membrane. Conversely, the AA simulation in this study showed that a number of incorporated caprate and caprylate molecules were expelled during the simulation (Figure 3A). In this earlier CG study, unlike the present study, the PE molecules were initially placed outside the membrane and the ability of PE molecules to be incorporated into the membrane was investigated. To shed light on whether the difference in the results between our results presented here and those reported in the work of Hossain et al.^37^ arises as a consequence of the initial placement of the PE molecules, we here performed CG MD simulations, matching the AA simulation setup with PEs (caprate, caprylate, and SNAC only) initially inserted into the membrane. In these simulations, we observed that only one and two expulsion events occurred for caprate and caprylate, respectively. At the end of the 4-μs long CG simulations, all the caprate and caprylate molecules were still found inside the membrane. However, for SNAC, a number of expulsion events occurred and only about 50% SNAC molecules remained in the leaflet where they were initially inserted (Figure S2).

**Figure 4:**
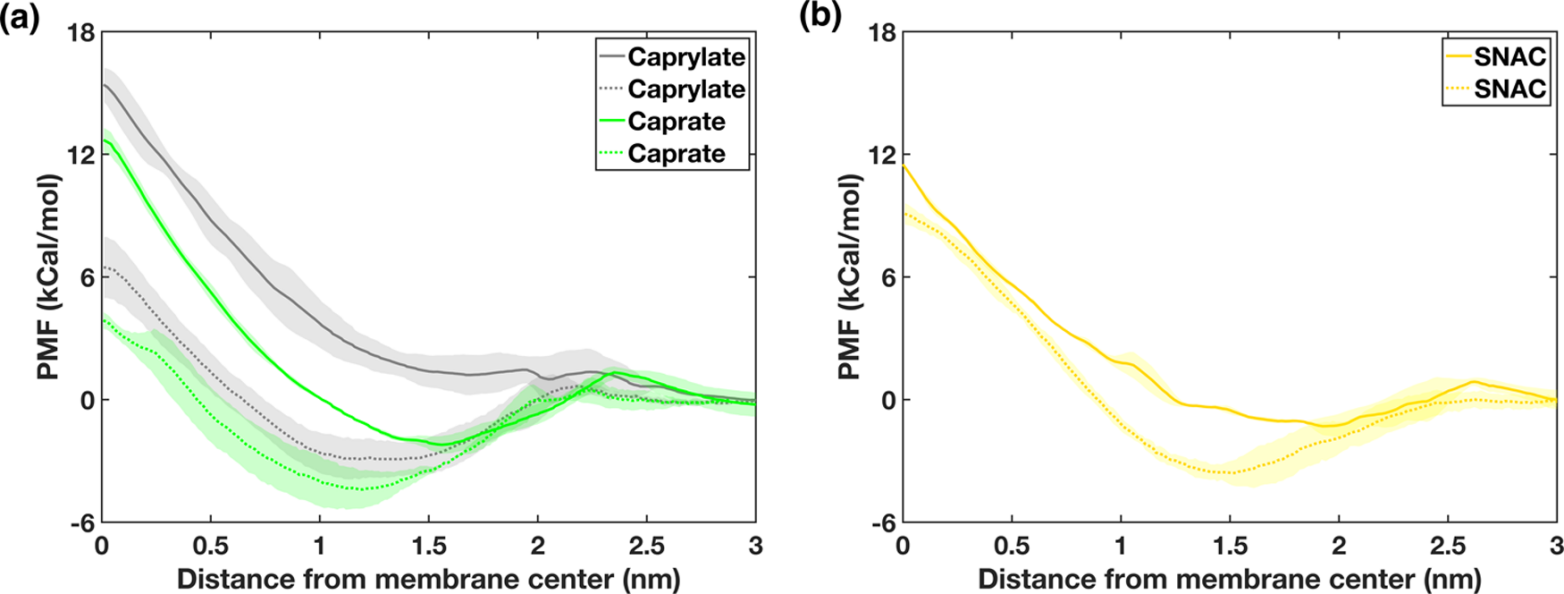
Potential of mean force (PMF) profiles obtained using all-atom (solid lines) and coarse-grained (dotted lines) MD simulations for (a) caprate and caprylate and (b) SNAC depicting the energy required to pull each PE molecule from the membrane center to the aqueous phase. In the PMF profiles, the lines and shaded regions represent the means and standard deviations, respectively, of triplicate simulations.

To further investigate the differences between the AA and CG simulation results, we also performed US simulations using both AA and CG force fields to obtain the PMF profiles associated with the pulling of caprate, caprylate, and SNAC molecules from the membrane center to the water phase for each simulation resolution. The corresponding PMF profiles are presented in Figure 4. From the profiles, we determined the location of the energy minima within the membrane for each case (PE and AA/CG) and the free energy differences, ΔG, between the water phase (which was used as the point of reference in each case) and the energy minima inside the bilayer. Note that the bilayer leaflet thickness for pure POPC was 1.95 nm. Except for the AA PMF profile for caprylate, the energy minimum was located within the bilayer region of their PMF profiles. For the caprylate AA PMF profile, the global minimum was in the water phase instead (although the PMF in this case is quite flat). For each PE, the energy minima obtained from AA US simulation was both more shallow and closer to the membrane headgroup region when compared to the CG simulations. This indicates that the free energy associated with an expulsion event reduced in AA relative to the CG simulations, consistent with expulsion events for caprate and caprylate being observed more rarely in CG simulation. For SNAC, although the energy minimum was also lower in CG than AA, it was at the same time somewhat closer to the membrane headgroup region (∼1.5 nm from membrane center) compared to caprylate and caprate, making expulsion events more likely to be observed in both CG and AA simulations for SNAC.

To further quantify the perceived methodological differences for how caprylate, caprate, and SNAC interact with POPC membranes, we used the free energy from the PMFs to estimate the equilibrium ratio of PE molecules outside and inside of the membrane using the following equation (eq 1).
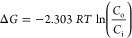
1Eq 1 can be rearranged to obtain the ratio of PE molecules outside and inside of the membrane,  (corresponding to the Δ*G* obtained from the PMF), as follows in eq 2:
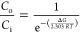
2Here, *R* is the ideal gas constant, *T* is the temperature, and *C*_i_ and *C*_o_ are the (number) concentrations of PEs inside and outside of the membrane, respectively.

The values of Δ*G* and *C*_o_/*C*_i_ are summarized in Table 2, with *C*_o_/*C*_i_ representing the ratio of PE molecules present outside or inside of the membrane at any given concentration. The *C*_o_/*C*_i_ value obtained from the AA US simulations suggests that approximately two times more caprylate molecules will be present outside of the membrane, and for caprate and SNAC, about 20 and 40% of the molecules will be expelled out of the membrane, respectively. This estimate qualitatively agrees well with our unbiased simulated result (Figure 3A), which shows that at 500 ns, two times more caprylate molecules are outside the membrane. For caprate and SNAC, about 30 and 40% of the molecules were expelled out of the membrane.

**Table 2:** Summary of the Umbrella Sampling Simulation Results Using All-Atom and Coarse-Grained Force Fields

	all-atom			coarse-grained		
	caprylate	caprate	SNAC	caprylate	caprate	SNAC
Δ*G* (kCal/mol)	0.99	–2.21	–1.30	–2.92	–4.40	–3.25
energy minima distance from the membrane center (nm)	2.05	1.56	1.90	1.38	1.18	1.52
*C*_o_/*C*_i_	2.01	0.21	0.40	0.13	0.04	0.10

Note that both AA and CG methods suggest that a certain amount of both caprate and caprylate molecules stayed incorporated into the POPC membrane. However, the difference between the number of expulsion events for caprate and caprylate molecules observed in the AA-method compared to CG-method is relatively large. We expect that because the CG methods use a significantly lower number of interaction sites compared to AA methods, the accuracy of CG methods to achieve such a detailed PE membrane interaction would be lower compared to the AA methods.^65^ Indeed, the interaction pattern of caprate and caprylate with the POPC membrane captured by AA methods agrees well with our previously published quartz crystal microbalance with dissipation monitoring (QCM-D) experimental results.^37^ The QCM-D experiments were utilized to estimate the amount of caprate and caprylate incorporated into the POPC membrane in the presence of FaSSIF. Those experiments showed that a higher amount of caprate molecules were incorporated into the membrane in the presence of FaSSIF compared to caprylate.

Overall, the results provided in this section indicate that PE chain lengths can significantly affect the degree of interaction with the membrane. Depending on concentration, two to three times more caprate molecules (Figures 3 and S1) are inserted into or interact with the membrane compared to caprylate. This finding agrees well with other literature studies in which it can be seen that the concentration required to increase the membrane fluidity decreases with the PE chain length.^66^ In addition to the chain length, the structural properties of the PE molecules can also affect their ability to interact with the lipid membrane. The presence of the salicylamide region in one end of the SNAC molecule reduces its ability to remain inside the membrane and in our systems, which likely contributes to the increase in the number of expulsion events for SNAC. Also, careful attention is needed in order to choose the force fields to study the PE interaction with the membrane using MD simulations. PMF profiles associated with the pulling of three different PEs showed different behaviors using AA and CG force fields. Although similar interaction patterns were observed in both AA and CG simulations for SNAC, the number of expulsion events for caprate and caprylate was underestimated in CG compared to the AA simulations.

### Changes in Membrane Structural Properties in the Presence of PEs

**Figure 5:**
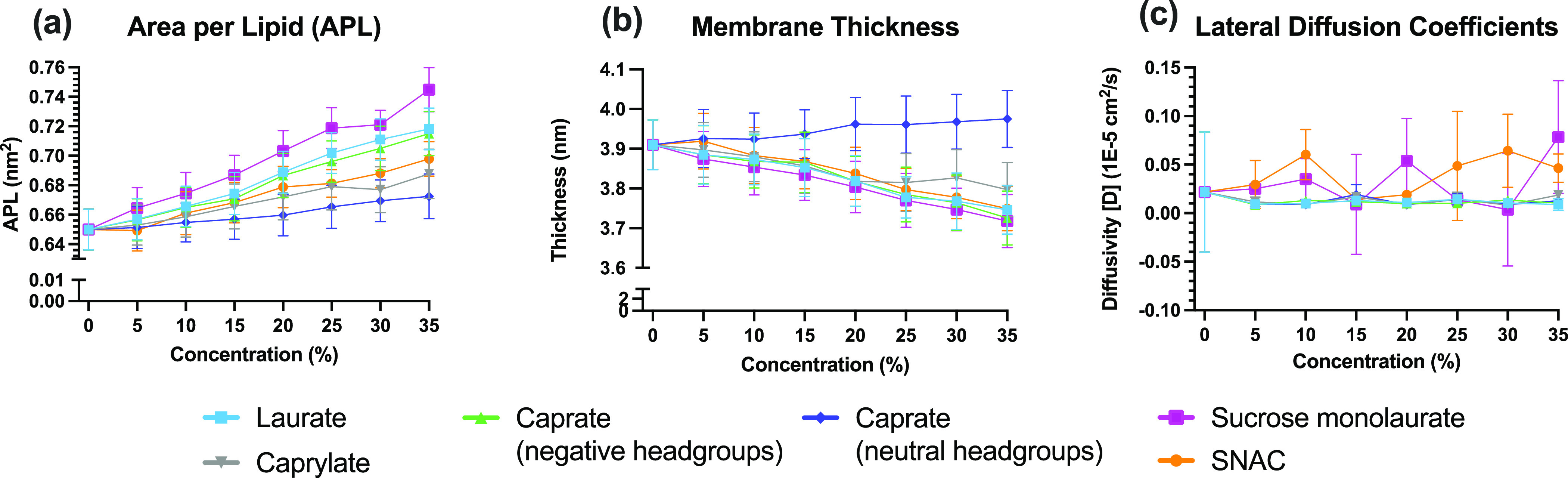
PE effect on membrane structural properties. The changes in different structural properties: (a) APL, (b) thickness, and (c) lateral diffusion coefficient (*D*_L_) for membrane POPC molecules calculated in the presence of different PEs at various number concentrations.

After quantifying the number of PEs that interact with the membrane, changes in membrane structural properties due to the presence of PEs in the membrane were investigated. To characterize the changes in membrane structural properties, we calculated the changes in APL, membrane thickness, order parameters (*S*_CD_) for POPC tails, and lateral diffusion coefficient (*D*_L_) at various concentration levels. The changes in APL, which provide information about the increase in the membrane area due to the presence of different PEs, are presented in Figure 5A. For each PE, an increase in APL with increasing concentration of PEs in the membrane can be seen. The APL for a POPC membrane without any PEs was determined to be approx. 0.65 nm^2^ per lipid. The experimental value reported by Kučerka et al.^67^ is 0.683 nm^2^ per lipid. Kučerka et al.,^68^ reported another APL value of pure POPC of 0.643 nm^2^ at 30 °C using neutron and X-ray scattering analysis. A third value that Klauda et al.^69^ reported is the NPT ensemble value obtained with Charmm36 from MD simulations of 0.647 nm^2^ per lipid. In our study, the maximum changes in APL were observed for sucrose monolaurate among different PEs at all concentration levels. For 35% sucrose monolaurate, there was a 14.6% increase in the APL value compared to the APL value of the membrane without any PEs (the pure POPC system). The changes in APL for negatively charged caprylate, caprate, and laurate followed the same order as their chain length, with higher increase in APL happening for PEs with longer carbon chains. This increase in APL can be assumed to be a direct consequence of the increased number of charged molecules in the bilayers, which would lead to more repulsive interactions between the PE and POPC molecules. This is in agreement with Langmuir monolayer experiments, which shows an increase in the area per molecule in a monolayer system by about 9% when pH is increased from 4 to 8.^70^ It might also be rationalized in light of the expulsion events described above for the PEs during the simulations where shorter-chain PEs are being expelled from the membrane to a larger extent, which in turn translates to a reduced impact on the changes of membrane APL. Caprate molecules with neutral headgroups showed the lowest increase in APL among the PEs investigated in this study. The flip-flop events of the neutral caprate molecules within the membrane means that molecules become more evenly distributed in both leaflets, leading to a lower increase in APL in this case. SNAC, when present at 35% concentration, also showed an increase of about 7%, compared to the pure POPC system without PEs. Note that a number of expulsion events also occurred for SNAC molecules during the simulations.

Changes in membrane thickness showed almost the opposite behavior compared to the changes in APL (Figure 5B), with the exception of neutral caprate. For each PE, there was a decrease in membrane thickness with the increasing PE % concentration. This decrease is mainly due to the presence of PE molecules near the headgroup region of the membrane, which tends to push the POPC molecules apart from each other. When this happens, packing of the lipid tails is disrupted, translated into a reduction of the effective length of the tails, reducing bilayer thickness. However, for the neutrally charged caprate, a slightly increasing trend in thickness was observed with increasing PE concentration. This is consistent with the increase in acyl chain order parameters seen for this particular case. Additionally, the location of the neutral caprate molecules was much deeper inside the membrane compared to the other fatty acid molecules that had negatively charged headgroups. We calculated the average distance, *d*, between the membrane center and the center of mass for caprate molecules (both negatively charged and neutral headgroups) in the membrane normal direction. The value of *d* was found to be 1.49 and 1 nm for the caprate molecules with negatively charged and neutral headgroups, respectively.

To further understand the changes in membranes’ structural integrity, we also calculated the lateral diffusion coefficient (*D*_L_) for membrane POPC molecules in the presence of different PEs at various concentrations, which are shown in Figure 5C. Unlike the other properties discussed above in this section, we did not see a clear trend of changes in *D*_L_ with the changes in PEs’ concentration. However, for most cases, the maximum *D*_L_ value was observed for SNAC. This is also due to SNAC’s location and orientation at the membrane surface. As SNAC mostly remains near the POPC headgroup region, it can induce more lateral movement of the POPC headgroup atoms and increase the *D*_L_ value of the POPC molecules. Sucrose monolaurate also showed higher *D*_L_ values compared to the other fatty acid PEs, which is mainly due to its relatively larger size.

**Figure 6:**
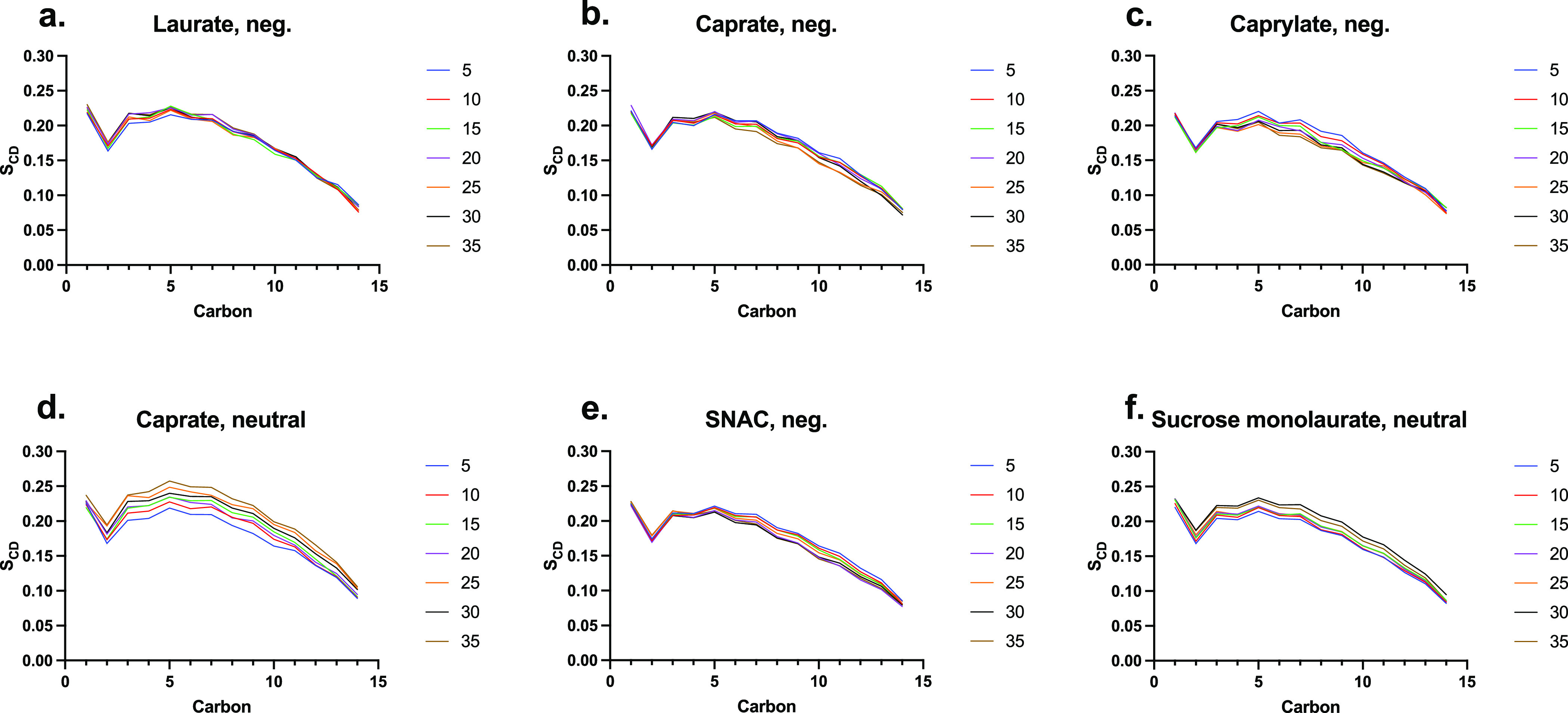
Carbon–deuterium order parameter for the sn-1 acyl chain of POPC (*S*_CD_), plotted for each PE in number concentrations: (a) negative sodium laurate, (b) negative sodium caprate, (c) negative sodium caprylate, (d) neutral sodium caprate, (e) negative SNAC, and (f) neutral sucrose monolaurate.

The changes in order parameter (*S*_CD_) values for the tails of membrane POPC are presented in Figure 6. The POPC tail order parameter can characterize the membrane structure, with a lower order parameter representing higher disruption within the membrane. For the negatively charged PEs, we observed slightly decreasing POPC tail *S*_CD_*-*values with increasing concentration, indicating a concentration-dependent PE effect on bilayer fluidity. The fatty acids with negatively charged headgroups showed a trend according to their chain length, with longer chain fatty acids causing less disruption compared to shorter-chain fatty acids. The neutral molecules show an opposite trend, most highly pronounced for neutral caprate. SNAC qualitatively follows the behavior of the other negatively charged PE concentration-induced changes to the order parameter, but the effect appears to be somewhat reduced. This is mainly due to the inability of SNAC molecules to remain incorporated into the membrane, as well as to penetrate deeper inside the membrane. Note that the value of *d* (the average distance between the center of the bilayer and the PE molecules) was found to be 1.9 nm for SNAC, which is relatively high compared to, for example, caprate molecules. To estimate the PE penetration ability inside the membrane, in addition to *d*, the average order parameter of the PE molecules themselves was also calculated and is presented in Figure S3. This analysis, with the average order parameter for SNAC found to be 20% lower compared to the caprate molecules, suggests that the SNAC molecules typically remain relatively parallel to the membrane surface and interact with the POPC headgroup region without inducing significant disruption in the membrane POPC tail region compared to the other PEs. Note that in this study, the membrane was composed of POPC only, which is abundantly available in the mammalian cells in general. However, phosphatidylethanolamine (POPE) and cholesterol are also typically present in the mammalian membrane. To what extent different PEs impact the membrane structural properties composed of various lipids and cholesterol demands further exploration. Another simplification made in the current study was the absence of any drug molecules. Therefore, only the absolute effect of the PEs on the membrane properties in the absence of drugs was obtained in this computational study. Neither different pH values nor the effect of sink conditions were taken into consideration. The relevant enzymes and the mucus normally present in the lumen were also missing, as was the possibility of exploring the paracellular routes.

The results provided in this section suggest that different chain lengths, sizes, and structural characteristics of PEs can affect the membrane structural properties. However, SNAC with the presence of a salicylamide region at the end of its chain can induce more lipid movement at the membrane surface without significantly changing the overall structural properties inside the membrane.

### Effect of PEs on the Water Permeation

**Figure 7:**
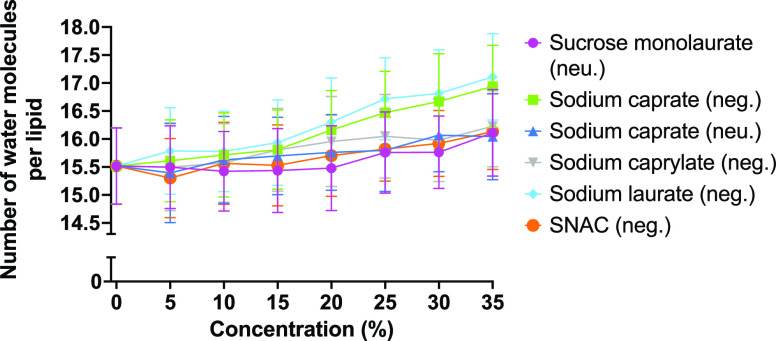
Number of water molecules per lipid (*n* = 128) near the POPC lipid tails at different PE concentrations, expressed as averages with standard deviation error bars. The largest increase in the amount of permeating water molecules can be seen for PEs with longer carbon chains and a negative charge. The two neutrally charged PEs, sodium caprate and sucrose monolaurate, along with the negatively charged SNAC show less of an increase, but are also able to induce water permeation over a pure POPC bilayer as a baseline.

The effect that the different PEs have on water permeation through the membrane was investigated by calculating the amount of water molecules present near the hydrophobic tails of the membrane. For each simulated system, the number of water molecules located near the carbon atoms of the POPC lipid tails was determined. There is a PE concentration-dependent increase in the number of water molecules near the lipid tails per lipid molecules (Figure 7). The maximum number of water molecules near the lipid tails was observed in the presence of laurate for all concentrations. For the negatively charged fatty acids, we can see an increasing trend in the number of water molecules near the lipid tails with increasing PE chain length, that is, sodium laurate > sodium caprate > sodium caprylate. This suggests that the presence of a higher number of PE molecules inside the membrane at a given concentration would increase the availability of water molecules near the hydrophobic lipid tails. Note that as shown in Figure 3A, the number of PE molecules that were present in the membrane at all concentrations followed the same trend.

In the systems with neutral caprate and negatively charged SNAC, the number of water molecules near the lipid tails were quite similar and slightly lower than that of the negatively charged fatty acids. We observed that the neutral caprate molecules typically interacted more with the membrane lipid tails compared to the negatively charged fatty acids. The average distance between the bilayer center and neutral caprate was 0.9 nm, in contrast to 1.52, 1.5, and 1.42 nm for negatively charged caprylate, caprate, and laurate, respectively. Therefore, neutral caprate restricted the water molecules’ contact with the membrane lipid tails compared to the other negatively charged fatty acids. On the other hand, as discussed in the earlier section, SNAC resides typically parallel to the membrane surface and interacts with the POPC headgroup region and thus can restrict the water molecules from going deeper into the model cell membrane. Sucrose monolaurate showed the lowest number of water molecules near the lipid tails for most of the PE concentrations used in the study. This is mainly due to the presence of the ester groups in the sucrose monolaurate molecules, which also typically resides near the membrane headgroup region and restricts the water beads to come in contact with the lipid tails.

Overall, our simulation results suggest that the PEs can increase the water molecules’ presence inside the membrane (near the hydrophobic tail region) with the increasing PE concentration. However, depending on the PE structural properties and their interaction pattern with the membrane, the extent of the increase can vary. At least for sodium caprate and laurate with a negative charge, the concentration at which they seem to start induce water permeation is around 15–20%, corresponding to about 70–100 mM (Table S1).

### Fractional Interactions between PEs and Membranes

**Figure 8:**
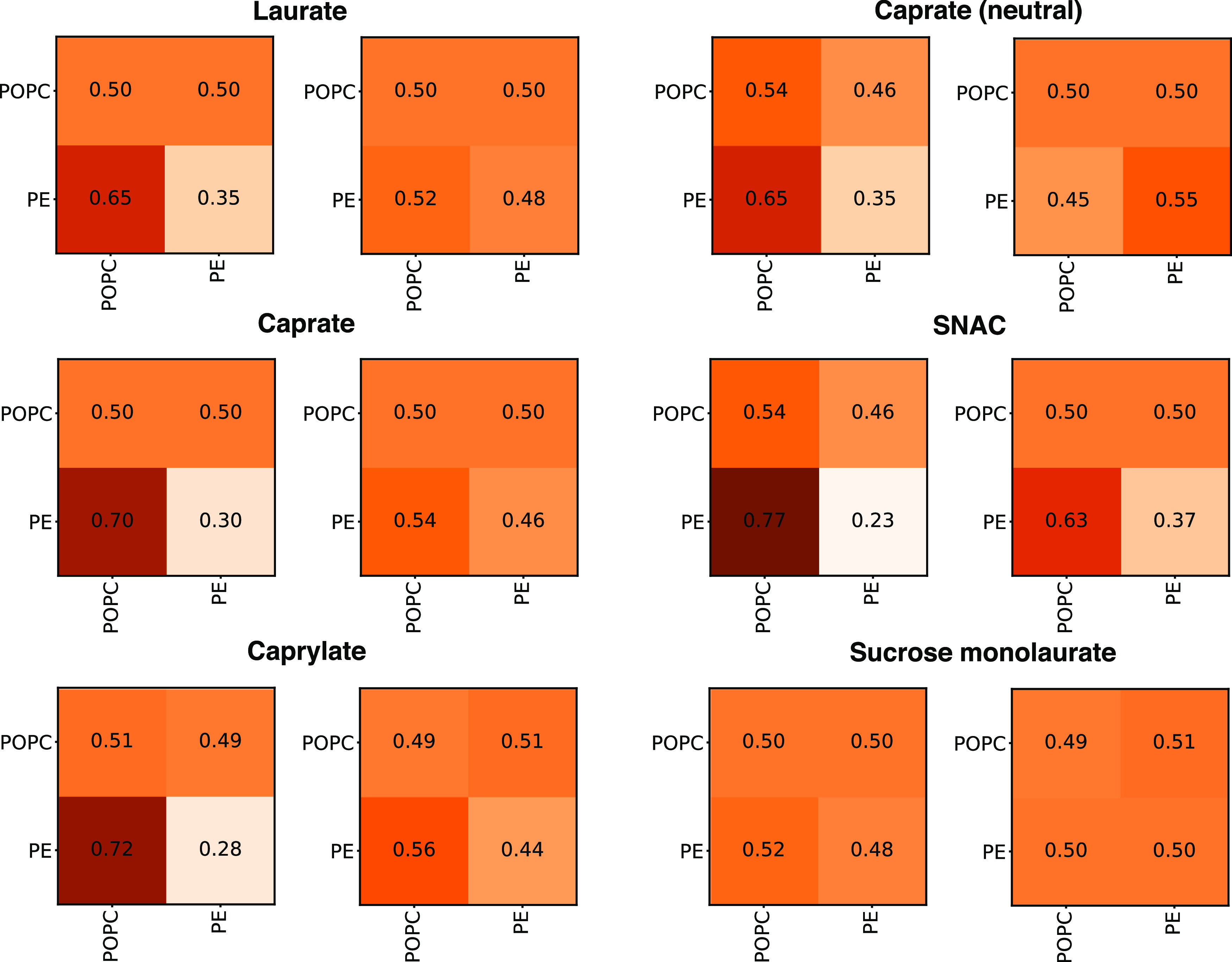
Fractional interaction matrix of different PEs and membrane POPC molecules. The matrix shows the fractional interaction as the relative number of contacts between PEs and/or POPC molecules compared to all other contacts. If the POPC/PE has more than one contact with another POPC/PE, this interaction is only counted once. Two molecules are defined as being in contact if the distance between the headgroup beads is less than 0.9 nm. The left panel represents 5% PE concentration, and the right panel represents the PE concentration of 35%. The coloring scheme goes from beige to dark brown, representing fractional interaction values closer to zero and one, respectively.

**Figure 9:**
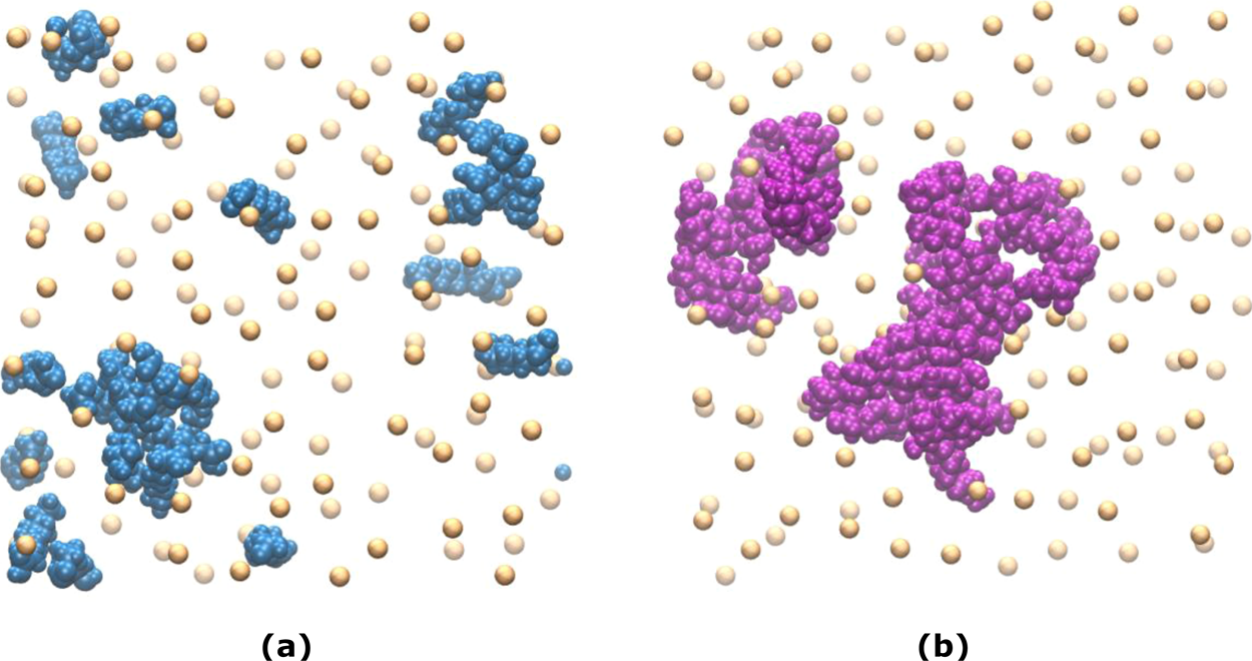
Formation of PE aggregates within the membrane. Top view snapshots showing the Pes with neutral headgroups: (a) sodium caprate and (b) sucrose monolaurate formed aggregates within the membrane surface. The images represent both the upper and the lower leaflets. The paler spheres are the phosphate headgroups of the POPC molecules in the lower leaflet (in both a and b), and the paler PE molecules in (a) are the caprate molecules in the lower leaflet. The aggregate in the lower left corner in (a) and both the aggregates in (b) are of interest.

To better understand the interactions of the PEs that remain in the inserted leaflet during the simulation with the membrane lipid molecules, we calculated the fractional interactions between them for all simulated systems. These are shown in Figure 8 for different PEs at 5 and 35% concentration levels. At 5% PE concentration, the results indicate that PEs interact preferably with the POPC molecules with >50% of total contact between PE-POPC for each PE. However, at 35% concentration, the PE–PE contact frequency increases for each PE, indicating formation of larger clusters of PE molecules, which was also observed visually in the simulations. At 35% concentration, caprate with neutral headgroups showed the highest percentage of PE–PE contact (with 55%), which suggests that the neutral caprate molecules prefer to interact with themselves within the membrane. PE–PE contact of sucrose monolaurate became 50% at 35% concentration. Figure 9 shows the presence of neutral PE aggregates within the membrane. Although the PE–PE fractional contacts for other charged PEs increase at 35% concentration compared to 5%, PEs still interact preferably with the POPC molecules with >50% of total contact between PE and POPC. Therefore, we did not observe any aggregates of negatively charged PEs during the simulations. SNAC showed the lowest PE–PE contact percentage among the PEs, which is again mainly due to their location and orientation within the membrane. This can be taken as another indication that higher concentrations of SNAC are required to induce membrane permeation-enhancing effects compared to, for example, sodium caprate which was also observed in the in vitro studies of Twarog et al.^19^

To better understand at which concentration the PE–PE contact becomes higher than 50% of total contact, we calculated the fraction contacts for the PEs with neutral headgroups at all concentrations, which are shown in Figure S5. We observed that at 10% PE concentration, the percentage of PE–PE contact becomes higher than 50% of total contact for caprate with neutral headgroups. However, sucrose monolaurate with only 35% concentration showed PE–PE contacts equal to 50% of total contacts. The results presented here suggest that PEs’ charged state plays the most important role in their contact interaction pattern within the membrane. Neutral PEs interact favorably with themselves and might form occasional aggregates within the membrane. The fact that PE molecules at some concentration seem to change from interacting preferably with surrounding POPC lipids to instead aggregate is interesting. Assuming that the simulations reflect the actual in vivo situation, it would provide a general molecular-level understanding of the mechanisms behind membrane disruption and loss of integrity that is often associated with high concentrations of PEs.^71,72^

## Conclusions and Future Plans

In this study, we used AA MD simulations to investigate the impact of six different PEs on the structural and dynamical properties of the model cell membrane. The simulation results obtained in our study indicate that PEs can impact the membrane structural properties in a concentration-dependent manner, that is, an increased PE concentration can induce higher membrane leakiness. MCFAs with shorter-chain lengths, such as caprylate, was observed to have a more dynamic interaction pattern with the cell membrane. The number of expulsion events of caprylate is relatively higher compared to MCFAs with a longer chain length (more than 10 carbon atoms). On the other hand, MCFAs with a relatively longer chain length, tend to remain inside the membrane once incorporated and therefore have a higher ability to disturb the model POPC membrane. The MCFAs’ ability to disrupt the cell monolayer as a function of their chain length was also observed by Brayden et al.^73^ We also observed that, in addition to the chain length, other structural characteristics of the PEs also impact their interaction pattern, that is, the presence of the salicylamide region in SNAC increased their number of expulsion events from the membrane. Moreover, this study also confirms that neutrally charged PEs, that is, caprate, can demonstrate flip-flop behavior within the membrane leaflets. Neutrally charged PEs, that is, caprate and sucrose monolaurate, also have the tendency to self-aggregate to a higher degree as compared to charged MCFAs.

In conclusion, we have presented how MD simulations can be used to understand the molecular-level interactions between a set of molecules that can be used to enhance permeability of orally administered drugs. In doing this, we in addition shed light on some methodological differences that are important to be aware about. A possible natural future step is to extend these studies to only include the active substance itself, such as a peptide therapeutic. It would also be interesting to attempt to model the influence of PE molecules on the drug permeation process in a kinetic framework, allowing for the determination of permeation rates under different conditions.

## Supporting Information Available

Variation of PE molecules with time that remains incorporated into the membrane leaflet where they were initially inserted; interactions of caprate, caprylate, and SNAC (all with negative charges) with the POPC membrane using CG MD simulations, snapshot of the initial and final system configurations of the system with 35% concentrations of PEs; average order parameter of different PE molecules; fractional interaction matrix of caprate (with neutral headgroups) and sucrose monolaurate with membrane POPC molecules at different PE concentrations; and detailed description of the simulated systems with various PE concentrations used for each PE (PDF)
