# Supplementary material for: In Silico-Based Experiments on Mechanistic Interactions between Several Intestinal Permeation Enhancers with a Lipid Bilayer Model
Source: Mol Pharm. 2021 Dec 16;19(1):124–37. doi: 10.1021/acs.molpharmaceut.1c00689 (PMC8728740; doi:10.1021/acs.molpharmaceut.1c00689)
Supplement: Supplementary file 1 — mp1c00689_si_001.pdf [file mp1c00689_si_001.pdf]

In silico-based experiments on mechanistic interactions between several intestinal permeation enhancers with a lipid bilayer model.

Authors: Rosita Kneiszl,[†,‡,§] Shakhawath Hossain[†,‡,§] and Per Larsson*[,†,‡]

[†]Department of Pharmacy, Uppsala University, Husargatan 3, 751 23 Uppsala, Sweden

[‡]SweDeliver, Uppsala University, Husargatan 3, 751 23 Uppsala, Sweden

[§]Equal contribution: analyses and writing

*Corresponding Author

Table of contents:

List of Figures	Page number
Supplementary Figure 1: Variation of PE molecules with time that remains incorporated into the membrane leaflet where they were initially inserted	2
Supplementary Figure 2: Interactions of caprate, caprylate and SNAC (all with negative charges) with POPC membrane using CG-MD simulations. Snapshot of (a) the initial and (b) final system configurations of the system with 35% concentrations of PEs were added.	3
Supplementary Figure 3: The average order parameter of different PE molecules	4
Supplementary Figure 4: Fractional interaction matrix of caprate (with neutral headgroups) and Sucrose monolaurate with membrane POPC molecules at different PE concentration	5

List of Tables	Page number
Supplementary Table 1. A detailed description of the simulated systems with vaious PE concentrations used for each PE.	6

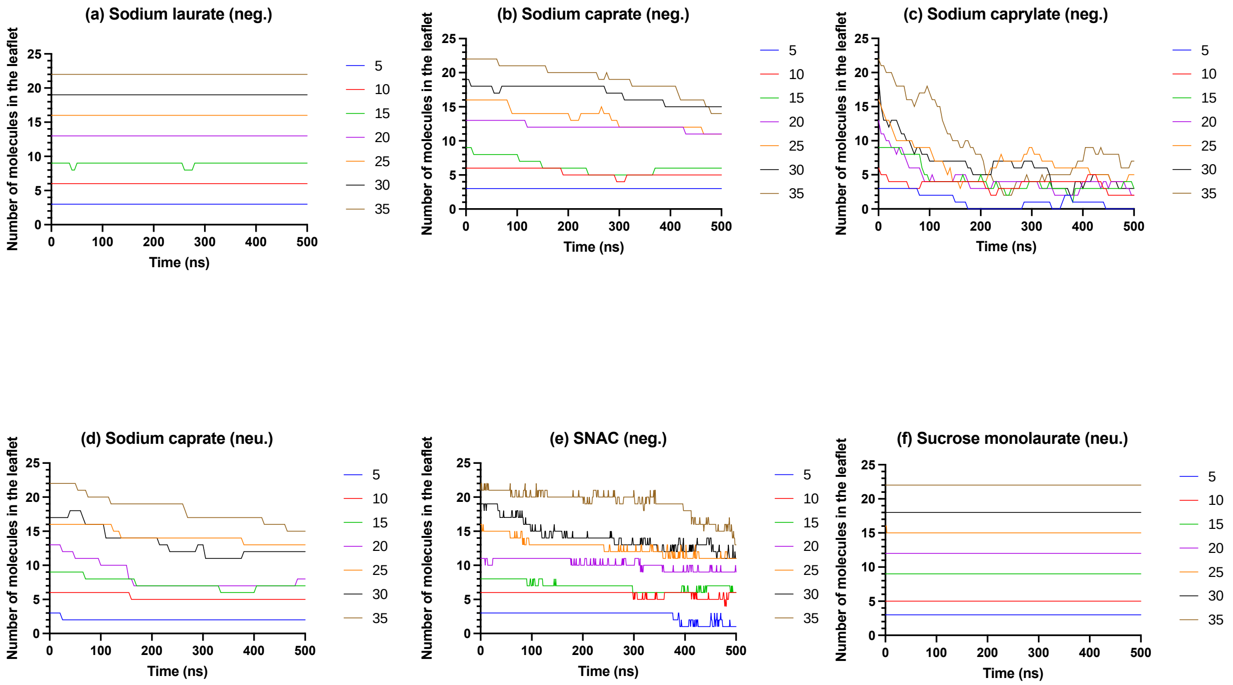

Supplementary Figure 1. The variation of the number of PE molecules over time that remains incorporated into the membrane leaflet in which they were initially inserted. All PE molecules except neutral sodium caprate did expel from the membrane. Neutral sodium caprate (d) did translocate within the membrane (flip-flop). Figures (a) neg. sodium laurate, (b) neg. sodium caprate, (c) neg. sodium caprylate, (d) neutral sodium caprate, note that all of the changes in the number of molecules is due to translocation within the membrane, so called flipflop events, from the “apical” side to the “basolateral” side of the model membrane, thus no expulsion events, (e) neg. SNAC (f) neutral sucrose monolaurate.

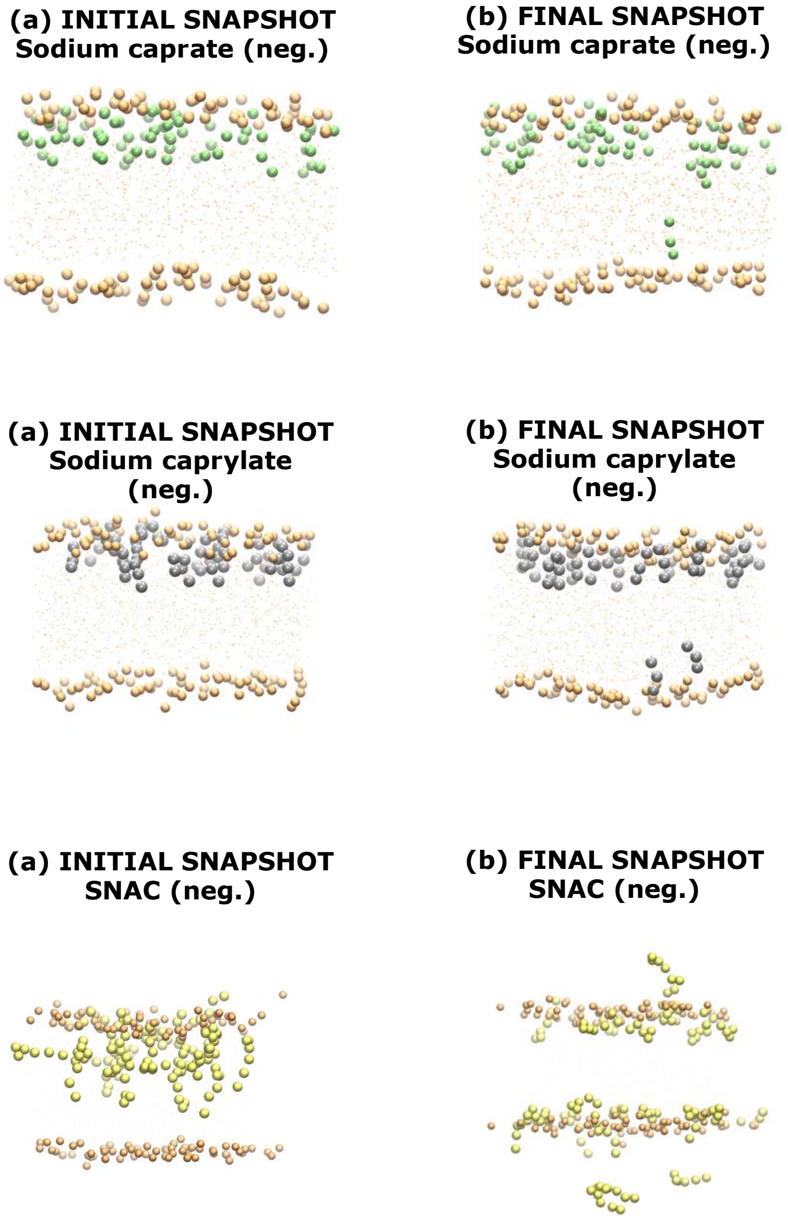

Supplementary Figure 2. Interactions of caprate, caprylate and SNAC (all with negative charges) with POPC membrane using CG-MD simulations. Snapshot of (a) the initial and (b) final system configurations of the system with 35% concentrations of PEs were added.

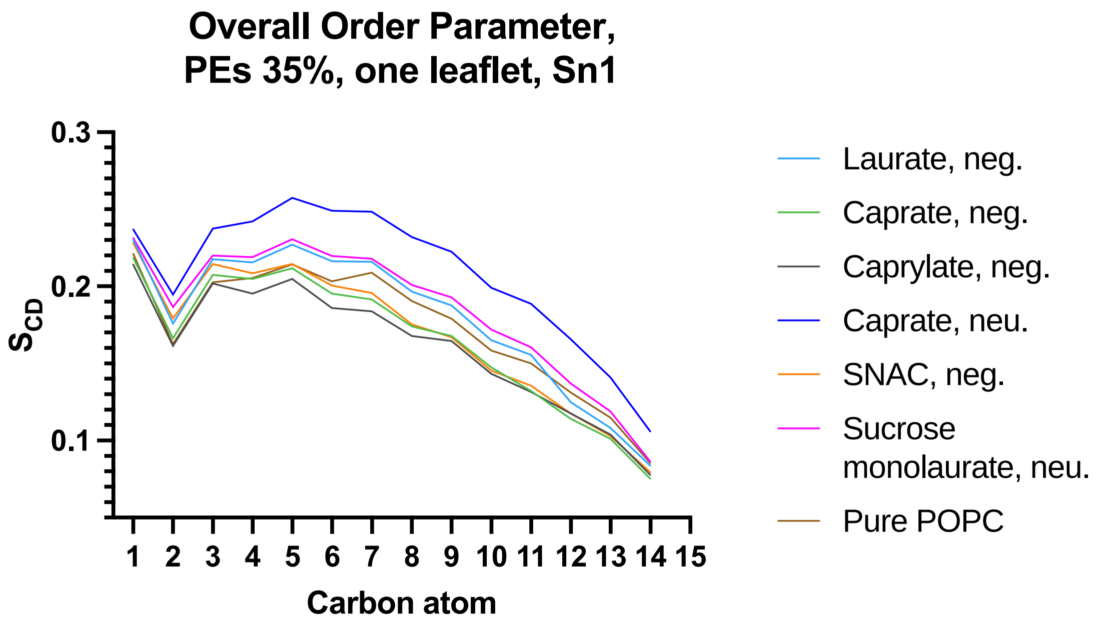

Supplementary Figure 3. The average order parameter of different PE molecules.

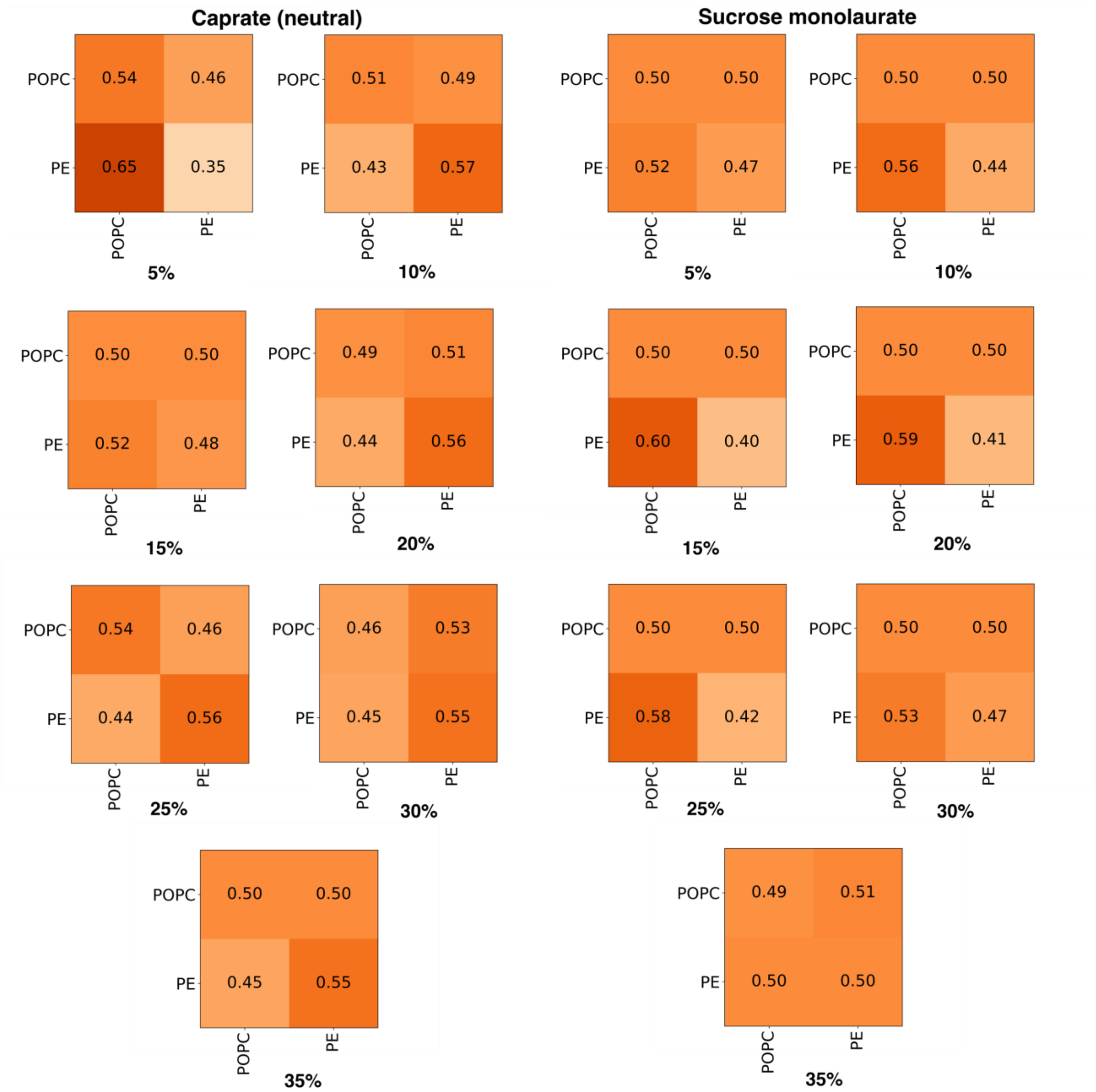

Supplementary Figure 4. Fractional interaction matrix of caprate (with neutral headgroups) and Sucrose monolaurate with membrane POPC molecules at different PE concentration.

Supplementary Table 1. A detailed description of the simulated systems with vaious PE concentrations used for each PE.

System[I]	C[II] (%)	Number[III]	X^{IV} (nm)	Y^{IV} (nm)	Z^{V} (nm)	C (M)	C[VI] (mM)
**0**	**0**	**0**	6.45	6.45	4.93	0.00	0.00
**1**	**5**	**3**	6.48	6.48	4.93	0.024	24.1
**2**	**10**	**6**	6.52	6.52	4.93	0.048	47.7
**3**	**15**	**9**	6.55	6.55	4.93	0.071	70.7
**4**	**20**	**13**	6.60	6.60	4.93	0.100	100.5
**5**	**25**	**16**	6.65	6.65	4.93	0.122	122
**6**	**30**	**19**	6.67	6.67	4.93	0.144	144
**7**	**35**	**22**	6.72	6.72	4.93	0.164	164

[I]System 0 where no PEs were present was simulated only once. System 1 to 7 were replicated for each PE showed in Table 1.
[II]PE number concentrations (%)
[III]The number of PE molecules inserted in one leaflet in each system.
[IV]The average lengths of the axes at that particular concentration for all six different types of PE molecules.
[V]Half of the average value of the membrane thickness obtained from this study, and the water phase on one side of the bilayer. NB: not the total value of the z-axis.
[VI]The millimolar concentration.
